# Supplementary material for: Comparison of the Ecological Traits and Boring Densities of Aromia bungii (Faldermann, 1835) (Coleoptera: Cerambycidae) in Two Host Tree Species
Source: Insects. 2022 Jan 30;13(2):151. doi: 10.3390/insects13020151 (PMC8877986; doi:10.3390/insects13020151)
Supplement: Supplementary file 1 [file insects-13-00151-s001.zip › insects-1534725-supplementary.pdf]

List of changes

P1 L5, 37: We checked our names and affiliations.

P2 L9: We confirmed that all highlighted red words were acceptable.

P2 L12: We changed “2013” to “2012”.

P7 L2: We added the revised Figure 2.

P11 L5: These indicates the min.–max. range of the lifetime fecundity (number of eggs). We think readers understand that if they read the previous sentence.

P12 L20: The supplementary materials were mentioned in main text as suggested.

P12 L25: The individual contribution of each co-author was checked.

P12 L31We checked the funding data.

P12 L35: We provided information in Informed Consent Statement.

P12 P38: We checked acknowledgements.

P13 L11: We changed the description of reference No.5.
